# Revisiting Abdominal Pain in IBS: From Pathophysiology to Targeted Management with Alverine Citrate/Simeticone

**DOI:** 10.3390/jcm15020722

**Published:** 2026-01-15

**Authors:** Rodolfo Sacco, Antonio Facciorusso, Edoardo Giannini, Massimo Bellini

**Affiliations:** 1 Gastroenterology and Digestive Endoscopy Unit, Department of Surgical and Medical Sciences, University of Foggia, 71122 Foggia, Italy; 2 Gastroenterology Unit, Department of Experimental Medicine, University of Salento, 73100 Lecce, Italy; 3 Gastroenterology Unit, Department of Internal Medicine, Istituto di Ricovero e Cura a Carattere Scientifico (IRCCS) Ospedale Policlinico San Martino, University of Genova, 16126 Genova, Italy; 4 Gastroenterology Unit, University of Pisa, 56124 Pisa, Italy

**Keywords:** abdominal pain, alverine citrate, gut–brain axis, irritable bowel syndrome, simeticone, visceral hypersensitivity, visceral pain

## Abstract

Abdominal pain is the cardinal symptom of irritable bowel syndrome (IBS) and the primary determinant of disease burden and healthcare utilization. Despite its diagnostic centrality and high prevalence across all IBS subtypes, effective management remains a clinical challenge. This narrative review explores the pathophysiological mechanisms underlying IBS-related pain, emphasizing the role of visceral hypersensitivity, altered brain–gut communication, and luminal factors such as gas and distension. We examine current guideline recommendations, real-world treatment patterns, and evidence supporting both pharmacological and non-pharmacological interventions. Particular focus is placed on the fixed-dose combination of alverine citrate/simeticone, which targets both motor and sensory pathways. Mechanistic studies demonstrate its smooth muscle relaxant, antinociceptive, and anti-inflammatory actions. Clinical trials support its efficacy in reducing pain, improving quality of life, and lowering healthcare resource use. Despite these advances, several unmet needs remain, including subtype-specific treatment strategies, mechanistic biomarkers, and broader access to integrated care. The review concludes with a call for more personalized, mechanism-based approaches to pain management in IBS, with alverine citrate/simeticone offering a pragmatic option within this evolving therapeutic framework.

## 1. Introduction

Irritable bowel syndrome (IBS) is a chronic and prevalent disorder of gut–brain interaction, defined by recurrent abdominal pain associated with changes in stool frequency and/or form, in the absence of structural abnormalities. The Rome IV diagnostic criteria emphasize abdominal pain—present at least 1 day per week over the past 3 months—as the essential feature of IBS, replacing the more ambiguous term “discomfort” used in earlier versions [1,2].

This conceptual shift aligns with clinical evidence showing that abdominal pain is not only diagnostic but also the most burdensome symptom reported by patients. In a large international web-based survey, over 90% of IBS patients cited abdominal pain, bowel dysfunction, and bloating as the most distressing features, with pain most strongly correlating with severity and poor quality of life [3]. More recent data from Canada and Italy confirm that moderate to severe pain is reported by 60–70% of individuals with IBS, particularly among women and younger adults [4,5]. Pain intensity also drives healthcare utilization, diagnostic testing, and the likelihood of psychiatric comorbidity, especially anxiety and depression [2,6].

Despite regional differences in the expression and reporting of abdominal symptoms, the global prevalence of IBS remains high. A meta-analysis of over 400,000 individuals found a pooled prevalence of 9.2% using Rome III criteria, which decreased to 3.8% with the more restrictive Rome IV definition [7]. In Italy, IBS with constipation predominance (IBS-C) appears to be the predominant subtype, though symptom overlap is common and pain severity frequently spans across subtypes [5]. These findings underscore the need for consistent diagnostic frameworks that account for cultural and linguistic differences in symptom expression. This is particularly important for pain, which may be reported variably as discomfort, bloating, or fullness [7].

The pathophysiology of abdominal pain in IBS is multifactorial, involving both peripheral and central mechanisms. Key contributors include visceral hypersensitivity (VH), altered motility, immune activation, gut dysbiosis and aberrant brain–gut communication [6,7,8]. These interacting pathways contribute to pain chronicity and variability, making management particularly challenging.

Although several pharmacological and non-pharmacological therapies are available, treatment remains largely symptom-driven. Antispasmodics and neuromodulators are recommended for pain relief [2,9,10]. However, many patients continue to experience suboptimal relief, pointing to the need for better targeted interventions.

Among current options, the fixed-dose combination of alverine citrate, a musculotropic antispasmodic, and simeticone, an anti-foaming agent, has emerged as a potentially effective strategy for addressing IBS-related abdominal pain and associated bloating [11,12]. The complementary mechanisms of these agents offer mechanistic and clinical synergy, particularly relevant for pain-predominant phenotypes.

This narrative review will explore the epidemiology, mechanisms, and management of abdominal pain in IBS, with particular focus on the clinical benefits and evidence supporting alverine citrate/simeticone combination as a pain-modulating treatment.

## 2. Methods

For this narrative review, we conducted a targeted literature search using PubMed, Scopus, and Google Scholar, focusing on publications from 2000 to 2025. The search terms included “irritable bowel syndrome,” “abdominal pain,” “visceral hypersensitivity,” “gut–brain axis”, “quality of life”, “alverine citrate,” and “simeticone.” Priority was given to systematic reviews, randomized controlled trials, clinical guidelines, and high-quality observational studies. Relevant articles were also identified through manual reference list screening of key publications. Only English-language articles were considered. The most recent and relevant data were integrated to provide a comprehensive and evidence-informed overview of the topic.

During the preparation of this manuscript/study, the authors used Academic Scientific Writer, a GTP of ChatGTP 5.0 for the purposes of reference styling. The authors have reviewed and edited the output and take full responsibility for the content of this publication.

## 3. Pathophysiology of Abdominal Pain in IBS

**Figure 1:**
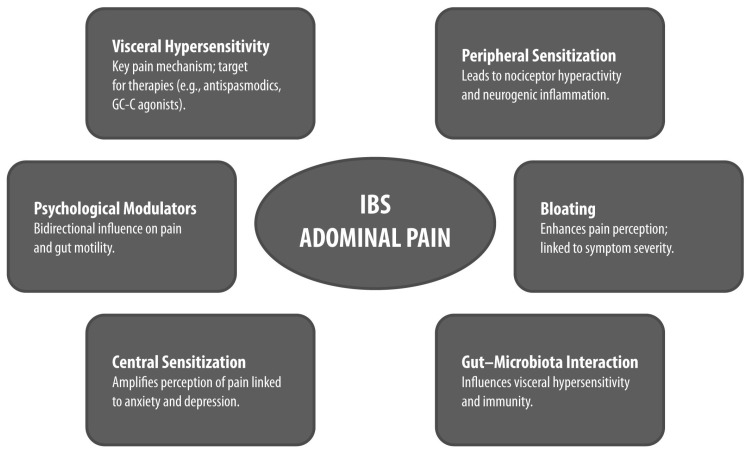
Pathophysiological and Clinical Features of Abdominal Pain in IBS. Conceptual diagram summarizing the pathophysiological and clinical features of abdominal pain in IBS. Each bubble highlights a key mechanism or modulator, with emphasis on clinical relevance and subtype differences GC-C: guanylate cyclase-C; IBS: irritable bowel syndrome.

Abdominal pain in IBS arises from complex and multifactorial mechanisms involving both peripheral and central sensitization within the gut–brain axis (Figure 1). Among these, VH has been consistently recognized as the defining pathophysiological substrate for pain in IBS and a major differentiating feature from organic gastrointestinal diseases [13,14].

### 3.1. VH and Peripheral Mechanisms

VH refers to an exaggerated pain response to normal or mildly noxious stimuli in the gastrointestinal tract. It is present in up to 60–90% of patients with IBS, particularly those with heightened symptom severity [8,13,15]. Mechanistically, VH results from sensitization of visceral afferent pathways, starting at the level of intestinal nociceptors. These nociceptors, located in the mucosa, serosa, and muscular layers, are activated by mechanical stretch, distension, and chemical mediators such as serotonin, histamine, and proteases [14].

Increased permeability of the intestinal mucosa in IBS may permit luminal antigens and microbial products to interact with underlying sensory nerves and immune cells, amplifying pain signals. For instance, mast cell proximity to enteric nerves has been associated with heightened pain perception and mucosal immune activation [13]. Several ion channels, including TRPV1 and Piezo2, are upregulated in IBS and contribute to mechanosensitive transduction, linking luminal stimuli to visceral pain [14].

Luminal dysbiosis, particularly shifts in histamine-producing bacteria, has also been implicated in modulating nociception via immune–neural pathways. These microbial changes may facilitate low-grade inflammation and drive serotonin and cytokine release, which in turn influence nociceptive signaling [8,15]. Notably, serotonin, synthesized by enterochromaffin cells, modulates both motility and pain sensitivity through activation of 5-HT3 and 5-HT4 receptors, further emphasizing the overlapping roles of sensory and motor dysfunction [8,13,15].

Lastly, bloating, reported in more than 80% of IBS patients, is strongly associated with pain and overall symptom severity. It is driven by multifactorial mechanisms, including VH, peripheral and central sensitization, altered microbiota composition, abnormal motility, and impaired gas handling [16].

### 3.2. Central Mechanisms and the Gut–Brain Axis

Beyond the gut, alterations in central pain processing play a key role in chronic visceral pain in IBS. Functional neuroimaging studies demonstrate abnormal activation in brain regions involved in pain perception, such as the anterior cingulate cortex, prefrontal cortex, thalamus, and insula, in response to colorectal distension in IBS patients [8,15]. These findings point to disrupted descending pain inhibition, which normally serves to dampen peripheral nociceptive input [14].

Stress and psychological comorbidities such as anxiety and depression further exacerbate pain processing by modulating hypothalamic–pituitary–adrenal (HPA) axis responses and neuroimmune signaling, reinforcing the bi-directional dysregulation of the microbiota–gut–brain axis [8,13,15].

### 3.3. Clinical Characteristics of Abdominal Pain in IBS

Clinically, IBS-related pain is typically intermittent, crampy, and diffuse, but varies widely among individuals. It often localizes to the lower abdomen and is closely linked with bowel movements, frequently improving after defecation or associated with stool passage [17]. Pain may worsen with stress, meals, or hormonal fluctuations, and is commonly accompanied by bloating, urgency, or a sensation of incomplete evacuation.

Patients with more severe or frequent pain often exhibit coexisting extra-intestinal symptoms, including fatigue and sleep disturbances, highlighting the multisystemic nature of VH [8,15,17].

### 3.4. Differences Among IBS Subtypes

Emerging evidence suggests that the manifestation of abdominal pain differs across IBS subtypes. In a large population-based study using PROMIS pain domains, individuals with IBS-C reported significantly more frequent, bothersome, and diffuse abdominal pain than those with IBS with diarrhea predominance (IBS-D) or IBS with mixed bowel habits (IBS-M) [17]. IBS-M also showed a trend toward more widespread pain compared to IBS-D, but differences in severity were less pronounced.

These subtype-specific patterns may reflect underlying differences in visceral sensitivity, mucosal immune activation, and central pain modulation [8,15,17]. For example, IBS-C patients show increased activation in pain-relevant brain regions and a higher density of mucosal mast cells, potentially contributing to the more diffuse and intrusive nature of their abdominal pain [14].

## 4. Impact of Abdominal Pain in IBS

Abdominal pain is a defining symptom of IBS and contributes substantially to reductions in quality of life, emotional well-being, and the increased use of healthcare resources. Although IBS includes a broad spectrum of gastrointestinal complaints, several studies indicate that pain, more than stool form or frequency, plays a key role in shaping the patient’s perception of illness severity and daily impact [4].

A Canadian survey of over 2400 individuals with IBS reported that 63–70% experienced moderate to severe abdominal pain. This symptom was more closely associated than other gastrointestinal features with reduced disease control, lower treatment satisfaction, and impaired quality of life [4]. Similar findings were reported in an international internet-based study, where abdominal pain, bloating, bowel irregularities and dietary limitations were commonly reported. Among participants with severe IBS, the average number of days with activity restrictions exceeded 4 months per year, and 30% of patients were not working due to illness [18].

Data from Italy provide further support for the prominent role of pain in IBS-related burden. In the Association of Hospital Gastroenterologists and Endoscopists (AIGO) multicenter survey, which enrolled 677 patients across 26 gastroenterology centers, abdominal pain intensity was significantly higher in women than in men. Overall, 38.5% of participants had severe IBS according to the IBS Symptom Severity Score (IBS-SSS). Patients with constipation- and diarrhea-predominant subtypes reported more frequent and bothersome abdominal pain, while those with mixed symptoms showed more variable profiles [5].

Quality of life, assessed through the SF-12 questionnaire, declined progressively with increasing IBS-SSS scores. The impact was observed in both physical and mental components, with younger patients and women reporting lower mental health scores. Psychological distress was also common: pathological anxiety was present in 38.7% of women and 27.2% of men, while depression was less frequent but still notable, particularly in those with more severe gastrointestinal symptoms [5].

Notably, pain is often strongly associated with bloating, another highly bothersome symptom reported by more than 80% of IBS patients. Bloating severity has been shown to correlate with overall IBS symptom burden, higher pain scores, somatization, and depression. It also frequently coexists with comorbid pain conditions, such as fibromyalgia, suggesting shared underlying mechanisms and a compounded impact on quality of life [16].

Importantly, patients’ perceptions of care often did not align with symptom burden. In some reports, patients expressed dissatisfaction with physician engagement, particularly when pain was not explicitly addressed or when reassurance was perceived as dismissive. For instance, in a French patient association survey, only a minority felt their physician had sufficient knowledge of IBS, and a significant proportion had been told to “live with” their symptoms [19].

The complex interplay between abdominal pain and psychological symptoms, as well as its consistent association with reduced functioning and lower treatment satisfaction, highlights the need for individualized, symptom-oriented management strategies. While pharmacologic options may play a role, addressing pain in IBS often requires a combination of dietary, psychological, and behavioral approaches tailored to each patient’s profile and expectations [20,21].

Overall, abdominal pain is a frequent and clinically relevant manifestation of IBS. It is closely linked to symptom severity, healthcare use, and reduced quality of life, underscoring its relevance as a core target in clinical assessment and therapeutic decision-making.

## 5. Management of Pain in IBS: Current Guidelines and Implementation in Clinical Practice

### 5.1. Diagnosis: A Symptom-Based Strategy

All major international and national guidelines emphasize a positive, symptom-based diagnostic approach to IBS, particularly when abdominal pain is the predominant symptom. The Rome IV criteria, developed by the Rome Foundation, are the current reference standard. These require recurrent abdominal pain (on average, at least 1 day per week in the last 3 months), associated with defecation and/or changes in stool frequency or form, with symptom onset at least 6 months before diagnosis [9].

The American College of Gastroenterology clinical guideline [9]; the British Society of Gastroenterology guideline [22]; the joint United European Gastroenterology and European Society of Neurogastroenterology and Motility Consensus on IBS-D [23]; the Italian Multisociety Consensus developed by SIGE, SINGEM, AIGO, SIED, SIMG, SIGENP, and SIP [24]; and the recently published 2025 Seoul Consensus [25] all endorse this definition. Diagnosis should be established with minimal laboratory testing in the absence of alarm features. These include full blood count, C-reactive protein, fecal calprotectin, and serologic testing for celiac disease. Colonoscopy is reserved for patients over age 50 or those presenting with red flag symptoms.

Several guidelines, particularly the Italian and British ones, recommend assessing psychological comorbidities, such as anxiety and depression, due to their frequent coexistence with IBS and their impact on symptom severity [22,24]. Despite widespread consensus, the actual use of these recommendations in routine care varies significantly.

### 5.2. Non-Pharmacological and Pharmacological Management

Management of abdominal pain in IBS is multifaceted, involving strategies that address VH, motility disturbances, dysbiosis, and central pain processing. Current guidelines recommend an integration of both non-pharmacological and pharmacological modalities, often in a stepwise, patient-centered manner. In this section, we discuss the main recommendations from guidelines, while a more detailed review of the therapeutic evidence of specific treatment options is provided in the next chapter.

Besides dietary and lifestyle changes, which are considered the first step in managing pain in IBS, several guidelines (Italian, British and United European Gastroenterology/European Society of Neurogastroenterology and Motility) recommend antispasmodics, such as otilonium bromide, mebeverine, hyoscine and alverine citrate, as a first-line pharmacological option for intermittent or meal-related pain [22,23,24,25]. Notably, the Seoul Consensus highlights peppermint oil as an effective and safe antispasmodic for IBS pain [25].

Tricyclic antidepressants are recommended for chronic or sleep-disrupting pain due to their central and peripheral analgesic effects at low doses [9,22,24]. In contrast, selective serotonin reuptake inhibitors receive more cautious support. The American Gastroenterological Association offers only a conditional recommendation based on limited analgesic evidence, though Italian guidelines consider them for patients with coexisting mood disorders [24,26].

Other pharmacological agents, including eluxadoline (for IBS-D), 5-HT_3_ antagonists like ondansetron and alosetron, and rifaximin (for IBS-D with bloating), are variably recommended. Their efficacy on pain is limited and tends to be more pronounced in symptom-specific subgroups [9,22,23,24].

Microbiota-directed therapies, particularly probiotics, are increasingly explored for their role in modulating gut–brain interactions in IBS [27]. While Italian guidelines support their cautious use for global symptom relief, the evidence remains inconsistent and strain-specific, with better results for multi-strain formulations and those containing *Bifidobacterium* or *Lactobacillus* species [24]. The Seoul Consensus conditionally supports the use of probiotics due to the very low level of evidence [25], while the American guidelines suggest against their use [9].

In addition to probiotics, microbial metabolites such as butyric acid have demonstrated potential therapeutic value. In particular, butyric acid has been shown to reduce abdominal pain in patients with IBS, likely through its role in maintaining gut homeostasis, modulating enteric nervous system signaling, and exerting immunoregulatory and anti-inflammatory effects [28,29].

The low FODMAP diet, although primarily aimed at bloating and gas, also impacts the gut microbiota and is widely endorsed as a second-line intervention. In responsive patients, it may contribute to pain reduction by reducing fermentable substrate availability and luminal distention [24].

Psychologically directed therapies, such as cognitive–behavioral therapy, gut-directed hypnotherapy and other brain–gut behavioral interventions, are consistently supported by the American College of Gastroenterology, the British Society of Gastroenterology, and Italian guidelines. These approaches are particularly valuable for refractory or high-burden patients [9,22,24].

### 5.3. Implementation and Real-World Application

Despite widespread consensus, the application of these guidelines in clinical practice remains inconsistent [27,30,31]. A nationwide Italian survey of 235 general practitioners (GPs) found that more than half were aware of the Rome IV criteria; however, only three-quarters reported using them regularly. Furthermore, many GPs, particularly more experienced ones, continued to include symptoms, such as bloating, as diagnostic criteria; this highlights the persistence of traditional clinical heuristics over standardized criteria [27].

Referral to specialists was often prompted by management challenges or patient insistence rather than structured criteria. Younger practitioners tended to align more closely with guideline principles, while senior GPs more frequently emphasized motility-related etiologies for abdominal pain.

Globally, real-world prescribing data also reveal considerable deviation from guideline-based care. A recent study analyzing over 54,000 individuals with disorders of gut–brain interaction found that 14.8% of those diagnosed were on prescription pain medications. This was more than double the rate observed in those without such diagnoses. Geographic variation was notable, with usage ranging from 6.8% in Singapore to 25.7% in the UK and 15% in Italy [8]. The study identified multiple factors associated with pain medication use, including rural residency, psychological comorbidities, and increased healthcare visits.

These findings suggest that in practice, pain management in IBS often remains pharmacologically centered, with limited uptake of the multimodal, individualized approaches advocated in guidelines. Barriers include limited access to behavioral therapies, variability in clinician education, and systemic constraints within primary care. Bridging this gap will require better integration of guideline principles into daily practice, interdisciplinary collaboration, and improved access to non-pharmacological interventions.

## 6. Overview of Non-Pharmacological and Pharmacological Options for IBS Management

Given the breadth of available interventions for abdominal pain in IBS, the following section provides an overview of selected recent evidence and major review articles, with the aim of outlining practical and evidence-informed therapeutic categories relevant to clinical decision-making. A complete systematic review is beyond the scope of this paper.

**Figure 2:**
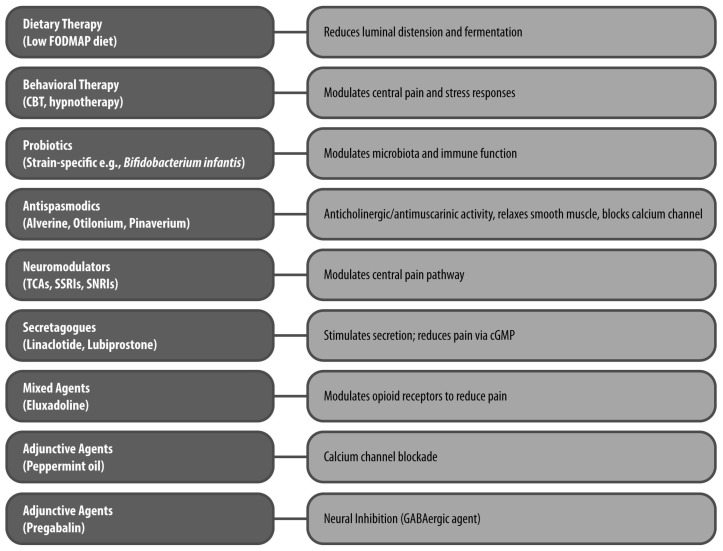
Therapeutic Classes and Mechanisms of Action in IBS Pain Management The figure illustrates key non-pharmacological (e.g., dietary and behavioral therapy) and pharmacological treatments (e.g., probiotics, antispasmodics, neuromodulators, secretagogues, mixed agents, peppermint oil and pregabalin) for IBS-related abdominal pain, highlighting their primary mechanisms of action. CBT: cognitive–behavioral therapy; cGMP: cyclic guanosine monophosphate; FODMAP: fermentable oligosaccharides, disaccharides, monosaccharides and polyols; SNRI: serotonin–norepinephrine reuptake inhibitor; SSRI: selective serotonin reuptake inhibitor; TCA: tricyclic antidepressant.

A summary of the non-pharmacological and pharmacological options, including treatment classes, mechanisms, and supporting evidence, is presented in Figure 2, while Table 1 outlines the results of the most relevant clinical trials and meta-analyses on key treatment strategies.

### 6.1. Non-Pharmacological Interventions

#### 6.1.1. Dietary Interventions

Dietary and lifestyle interventions are commonly recommended for patients with IBS [24]. One approach is Traditional Dietary Advice (TDA), which includes eating regular meals, adjusting fiber and fluid intake, and reducing consumption of fat, alcohol, and caffeine. Variants of TDA have been developed by organizations such as the National Institute for Health and Care Excellence (NICE) [48] and the British Dietetic Association (BDA) [49].

Another widely endorsed intervention is the low FODMAP diet, which restricts fermentable oligosaccharides, disaccharides, monosaccharides, and polyols. These short-chain carbohydrates are found in various foods, including certain fruits, vegetables, legumes, dairy products, and artificial sweeteners [24,32]. The low FODMAP diet is the most evidence-based dietary strategy for managing IBS symptoms, including abdominal pain. By reducing fermentable substrates, it minimizes gas production, luminal distension, and osmotic load, which are believed to contribute to symptom generation [50]. A recent network meta-analysis confirmed its superior efficacy over standard dietary advice for improving global symptoms, bloating, and abdominal pain [32].

Despite its effectiveness, the diet should be implemented under professional supervision due to risks of nutritional deficiencies, variable individual responses, and the complexity of its phased structure [14,50].

#### 6.1.2. Behavioral and Brain–Gut Therapies

Brain–gut behavioral therapies, such as cognitive–behavioral therapy, gut-directed hypnotherapy and multicomponent psychotherapy, are strongly supported by guidelines for patients with moderate to severe pain or when pharmacological therapy is insufficient [37]. These therapies target central modulation of pain perception and stress responses and have shown benefit even in refractory cases. In addition to psychobehavioral approaches, recent evidence highlights the potential of neuromodulatory techniques such as transcutaneous auricular vagus nerve stimulation (taVNS), particularly in IBS-C. This is supported by preclinical findings demonstrating vagally mediated effects on visceral sensitivity and gut motility in a mouse model of IBS-C [51], as well as by a randomized controlled trial showing improvements in abdominal pain, bowel habits, and psychological symptoms after 4 weeks of daily taVNS treatment [52].

#### 6.1.3. Probiotics

Select probiotic strains have been associated with reductions in abdominal pain and global IBS symptoms, although heterogeneity in formulations and study designs complicates direct comparisons [53]. While probiotics are generally safe, current recommendations favor strain-specific use guided by emerging evidence.

### 6.2. Pharmacological Interventions

#### 6.2.1. Antispasmodics

Antispasmodics are widely used to treat smooth muscle spasm and related pain. Notably, this drug class encompasses heterogeneous compounds with distinct mechanisms of action, which contribute to their varying efficacy and safety profiles. The main pharmacological groups include (i) anticholinergic/antimuscarinic agents, which inhibit gastrointestinal smooth muscle contraction primarily through muscarinic receptor blockade (e.g., dicyclomine, hyoscine, hyoscyamine, otilonium); (ii) calcium channel inhibitors, which prevent calcium influx into smooth muscle cells, thereby reducing muscle contractility (e.g., alverine, otilonium, pinaverium, trimebutine); and (iii) direct smooth muscle relaxants, which modulate both sodium and calcium transport to suppress duodenal and colonic contraction (e.g., dicyclomine, hyoscine, hyoscyamine, mebeverine) [54].

#### 6.2.2. Neuromodulators

Low-dose tricyclic antidepressants, such as amitriptyline and nortriptyline, are established second-line treatments for IBS-related pain. They act via modulation of descending inhibitory pain pathways and reduction in central sensitization [2,18]. Selective serotonin reuptake inhibitors and serotonin–norepinephrine reuptake inhibitors are also used, particularly in the presence of mood disorders, though their analgesic efficacy is more variable [42].

#### 6.2.3. Secretagogues and Mixed Mechanism Agents

Drugs such as linaclotide and lubiprostone, though primarily used for IBS-C, have demonstrated analgesic effects through activation of cyclic GMP pathways, reduction in VH, and improvement of bowel function [2]. Eluxadoline, approved for IBS-D, acts on opioid receptors and may reduce abdominal pain by modulating sensory signaling [55].

#### 6.2.4. Adjunctive and Investigational Agents

Other options include peppermint oil, with modest antispasmodic activity via calcium channel blockade, and GABAergic agents, such as pregabalin, which may reduce neural excitability. Histamine receptor antagonists, bile acid sequestrants, and neurokinin-2 receptor antagonists remain under investigation [41,55].

## 7. Alverine Citrate/Simeticone in IBS: Mechanistic Rationale and Clinical Evidence

The fixed-dose combination of alverine citrate and simeticone is used in the symptomatic management of IBS, particularly in patients with abdominal pain and bloating. The rationale for this formulation lies in the complementary pharmacological properties of its two components. Alverine citrate is a musculotropic spasmolytic that acts on intestinal smooth muscle and central pain pathways, while simeticone is a non-absorbable, inert anti-foaming agent that reduces intraluminal gas and distension. Together, they address motor, sensory, and luminal factors that contribute to IBS symptomatology, with particular relevance to pain modulation. Notably, the combination of alverine citrate and simeticone is recommended in several European guidelines [23,24] as a first-line option for managing abdominal pain and bloating in patients with IBS. Other guidelines, such as the 2025 Seoul Consensus, provide only a conditional recommendation due to low-certainty evidence [25], while the American guidelines do not mention this option [9].

### 7.1. Mechanism of Action

**Figure 3:**
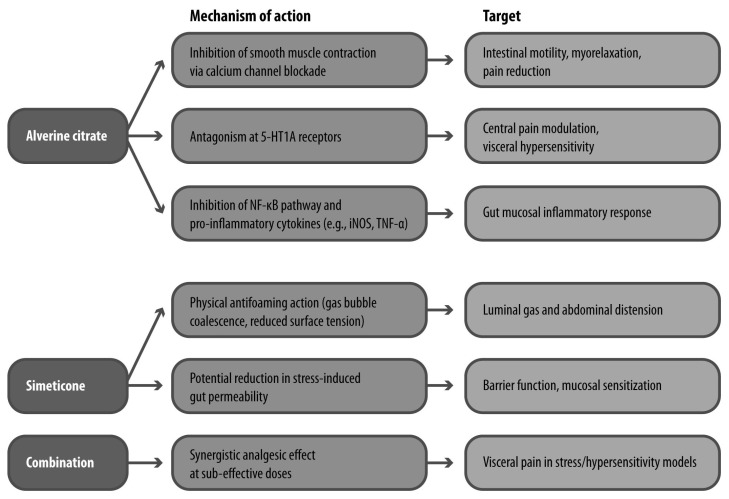
Mechanisms of Action of Alverine Citrate and Simeticone. 5-HT1A: 5-hydroxytryptamine receptor 1A; iNOS: inducible nitric oxide synthase; TNF-α: tumor necrosis factor alpha.

Alverine citrate acts at multiple levels of the gut and nervous system (Figure 3). Its antispasmodic activity has been demonstrated in vitro and in vivo, where it inhibits spontaneous and cholinergically stimulated contractions of gastrointestinal smooth muscle, likely via L-type calcium channel blockade. In preclinical models, it was shown to abolish contractile responses in the guinea pig ileum and attenuate colonic responses to vagal stimulation in rabbits, suggesting effects on both peripheral and enteric neural circuits [56].

Recent evidence has expanded its mechanistic profile to include anti-inflammatory properties. Alverine inhibits activation of the NF-κB signaling pathway and downstream inflammatory mediators such as inducible nitric oxide synthase and TNF-α. These effects are thought to be mediated through Src kinase inhibition, a mechanism potentially relevant to the low-grade inflammation associated with IBS [57].

Of particular interest is alverine’s interaction with serotonergic signaling. In a rat model of rectal hypersensitivity, alverine citrate significantly reduced the number of abdominal contractions following administration of 5-hydroxytryptophan, a serotonin precursor. The reversal of this effect by serotonergic agonists and alverine’s binding affinity for 5-HT1A receptors suggests that it acts as a functional antagonist at this receptor subtype, contributing to its antinociceptive properties [58].

Simeticone, while pharmacologically inert, disperses intraluminal gas bubbles and reduces abdominal distension, a symptom that often coexists with pain in IBS. It has also been shown in preclinical models to reduce intestinal permeability under stress conditions, suggesting a secondary protective effect against mucosal sensitization. When combined with alverine, simeticone may enhance its analgesic activity by reducing gas-induced mechanical stimuli and modulating barrier function (Figure 3) [59].

### 7.2. Preclinical Evidence

Animal studies support the independent and synergistic effects of alverine citrate and simeticone on VH. In rat models of rectal distension under chemical or mechanical stimulation, alverine reduced pain-related behavioral responses, particularly under inflammatory conditions. Systemic administration of alverine inhibited pain induced by serotonergic agents, and central administration confirmed its activity through 5-HT1A receptor antagonism [58].

In models of stress-induced colonic hyperalgesia, simeticone alone showed modest efficacy at high doses. However, when administered in combination with alverine at sub-effective doses, the reduction in visceral pain was significant. This potentiation effect is likely due to the agents targeting distinct yet complementary mechanisms: smooth muscle tone, sensory modulation, and luminal distension [59].

### 7.3. Clinical Evidence

The efficacy of alverine citrate/simeticone has been investigated in both controlled and pragmatic clinical settings (Table 2). In a multicenter, double-blind, randomized controlled trial, Wittmann et al. enrolled 409 adult patients fulfilling Rome III criteria for IBS with moderate to severe abdominal pain or discomfort. Participants received either alverine citrate/simeticone (60 mg/300 mg) or placebo three-times daily for 4 weeks, following a 2-week run-in period.

At week 4, the median VAS score for abdominal pain was significantly lower in the treatment group (40 mm vs. 50 mm; *p* = 0.047). The responder rate, defined as achieving at least 50% reduction in VAS pain score, was 46.8% in the alverine citrate/simeticone group versus 34.3% in the placebo group (*p* = 0.01), corresponding to a number needed to treat (NNT) of 8.

Early improvements were also seen by week 2 (VAS 51 mm vs. 59 mm; *p* = 0.02), with higher responder rates (27.9% vs. 17.2%; *p* = 0.01). A greater proportion of patients rated their global improvement positively in the treatment group (62.5% vs. 48.5%; *p* = 0.0001). Although the change in life impact scores favored alverine citrate/simeticone, the difference was not statistically significant (mean change: 0.97 vs. 0.76; *p* = 0.08) [11].

A pragmatic, open-label randomized study by Ducrotté et al. evaluated the use of alverine citrate/simeticone on demand over 6 months in primary care. A total of 436 patients with moderate to severe IBS (IBS-SSS 175–400) were enrolled and randomized to receive either on-demand alverine citrate/simeticone or usual care (primarily antispasmodics given on a continuous basis). The primary outcome was the change in IBS-specific quality of life (IBS-QoL).

At study completion, the mean improvement in IBS-QoL was significantly greater in the alverine citrate/simeticone group (13.8 ± 1.1 vs. 8.4 ± 1.2 points; *p* = 0.0008). Additionally, 58.6% of patients achieved a ≥50% reduction in IBS-SSS scores compared to 35.9% in the usual care group (*p* = 0.0001). Clinical remission (IBS-SSS < 75) was reached in 37.7% vs. 16.0% of patients, respectively (*p* < 0.0001). Pain improvement was reported by 76.1% of patients in the alverine citrate/simeticone group versus 59.2% in the control group (*p* = 0.0001), and bloating was improved in 76.6% vs. 57.0% (*p* < 0.0001). Notably, the reduction in IBS-SSS was observed across all IBS subtypes, but the difference between treatment groups was most pronounced in the IBS-C (*p* < 0.0001) and IBS-M (*p* = 0.0001) subgroups.

Among the secondary endpoints, IBS-QoL significantly improved, with a mean IBS-QoL score increase of 13.8 ± 1.1 points in the alverine/simeticone group versus 8.4 ± 1.2 in the usual care group (*p* = 0.0008), reflecting a 28.5% vs. 18.6% improvement from baseline (*p* = 0.04). Importantly, six QoL domains, including emotional health, mental health, sleep, energy, food-related concern, and social functioning, showed significantly greater improvements in the treatment arm, highlighting the broad impact of symptom control on patient well-being.

Fewer patients in the alverine/simeticone group required rescue medications in the alverine citrate/simeticone group, including analgesics (1.4% vs. 4.8%; *p* = 0.04) and psychotropic drugs (18.9% vs. 27.9%; *p* = 0.03). Healthcare utilization and daily treatment costs were also lower (mean €0.46 vs. €0.94; *p* < 0.0001), suggesting potential economic advantages [12].

In terms of safety/tolerability, the combination has demonstrated a favorable safety and tolerability profile across different studies. Ducrotté et al. [12] in the on-demand setting reported no significant difference in the incidence of adverse events between alverine/simeticone and placebo, with no treatment-related serious adverse events and only 2.2% of patients experiencing adverse events potentially related to alverine/simeticone. Similarly, Wittmann et al. [11] observed a favorable safety profile, with no significant increase in adverse events compared to placebo and no severe drug-related adverse events.

Together, these findings support the efficacy of alverine citrate/simeticone in improving abdominal pain and related symptoms in IBS, as well as improving patients’ QoL. The combination has demonstrated clinical benefit in both controlled and real-world environments, with favorable tolerability and potential to reduce adjunctive medication use and healthcare burden.

## 8. Reframing IBS Pain Management: Gaps, Opportunities, and the Role of Alverine Citrate/Simeticone

Despite decades of research, abdominal pain in IBS remains a clinically challenging and often frustrating symptom to manage. This review has outlined how pain, more than bowel dysfunction or bloating, defines the patient experience and drives healthcare engagement. However, therapeutic options remain variably effective, and their integration into routine care is inconsistent. Although international guidelines provide structured frameworks for diagnosis and treatment, evidence from clinical practice indicates that these are not always followed, particularly in primary care, where IBS is often still treated as a diagnosis of exclusion rather than as a positively identified functional disorder [2,27,31].

The available pharmacological strategies, ranging from antispasmodics and neuromodulators to secretagogues and serotonergic agents, offer modest benefit for many patients, yet a substantial proportion remains symptomatic even when guideline-recommended regimens are applied. This therapeutic gap reflects, in part, the complex and heterogeneous pathophysiology of IBS pain, which encompasses peripheral mechanisms, such as VH, altered motility and mucosal inflammation, as well as central contributors, including stress reactivity and dysregulated pain modulation [6,8,15]. Compounding the challenge is the fact that most therapies are not specifically tailored to pain pathomechanisms or IBS subtypes.

Emerging evidence points to distinct neurobiological profiles across IBS subgroups. For example, patients with IBS-C, particularly women, exhibit enhanced activation of brain regions such as the amygdala and thalamus, possibly reflecting heightened emotional salience and sensory amplification of visceral stimuli [8,15]. This aligns with earlier findings on VH and supports the idea that pain is not a uniform experience across patients, but rather a spectrum of overlapping neurogastrointestinal phenotypes. In this context, the limited personalization of IBS treatments represents a key unmet need and underscores the importance of mechanism-driven therapy.

Among the available therapies, several antispasmodics, including alverine, mebeverine, and peppermint oil, are used for symptom relief in IBS, often with overlapping indications. Each has a distinct mechanism of action and varying levels of evidence supporting efficacy. Within this therapeutic landscape, the fixed-dose combination of alverine citrate/simeticone offers an interesting case. Its dual mechanism, targeting both smooth muscle contractility and luminal gas retention, addresses two frequently co-occurring factors in IBS pain. Alverine’s antispasmodic, anti-inflammatory, and central antinociceptive effects have been shown in both preclinical models and in vitro studies, with particular relevance to serotonin-mediated hypersensitivity [57,58]. Simeticone, while inert, contributes by reducing abdominal distension and may enhance mucosal barrier function under stress [59].

Clinically, the efficacy of alverine citrate/simeticone has been demonstrated in both controlled and pragmatic settings. The randomized controlled trial by Wittmann et al. confirmed a statistically significant improvement in pain scores and global symptom perception over 4 weeks of treatment [11], while the real-world, 6-month trial by Ducrotté et al. showed improvements not only in pain and bloating but also in quality of life and healthcare utilization [12]. Moreover, although studies directly comparing the effects of alverine/simethicone across different IBS subtypes are lacking, some evidence suggests that this combination may be particularly effective in IBS-C and IBS-M, where bloating and postprandial cramping are common [12]. These findings are especially relevant in light of recent survey data showing that pain remains the symptom most strongly associated with treatment dissatisfaction and unmet expectations in patients with IBS, particularly those with IBS-C and overlapping psychological symptoms [10].

Still, key questions remain unanswered. Most notably, the current evidence base is limited by the absence of head-to-head trials comparing alverine citrate/simeticone with other recommended therapies, limiting definitive conclusions about its relative efficacy. Moreover, while the combination shows promise for symptom relief, further studies are needed to clarify its effects on underlying mechanisms, especially in relation to visceral sensitivity and neuroimmune interactions. Likewise, broader validation in more diverse and well-characterized patient populations, stratified by sex and psychological comorbidities, is essential to confirm generalizability.

Advancing the management of IBS pain will require a renewed focus on translational research, biomarker identification, and personalized therapeutic algorithms. Within this evolving framework, alverine citrate/simeticone may serve as a pragmatic and mechanistically grounded option for a subset of patients. Continued clinical investigation and integration into comparative effectiveness research will be crucial to refining its role in modern IBS care, especially including active comparisons with other active agents.

## 9. Future Directions

Despite progress in understanding and managing IBS-related abdominal pain, several unmet needs persist. There is growing interest in identifying reliable biomarkers to aid in diagnosis, subtype classification, and prediction of treatment response. Stratifying patients based on IBS subtypes or underlying mechanisms (e.g., visceral hypersensitivity, dysbiosis, or central sensitization) may enable more personalized therapeutic approaches. Additionally, from a clinical research perspective, future studies are needed to directly compare the efficacy and safety profiles of different treatment strategies. These should prioritize large-scale, head-to-head randomized controlled trials comparing current therapies with active comparators such as alverine citrate/simeticone, peppermint oil, otilonium or other antispasmodics or neuromodulators. Finally, mechanistic and translational studies linking pathophysiology to symptom profiles may help guide targeted interventions and improve the overall quality of IBS care.

## Figures and Tables

**Table 1:** Summary of Clinical Trial and Meta-analysis Evidence on Abdominal Pain Management in IBS.

Study	Patients (n)	Intervention	Key Findings on Abdominal Pain
Non-Pharmacological Interventions			
*Low FODMAP Diet*			
Meta-analysis (13 RCTs) [32]	944	Low FODMAP diet vs. habitual/sham/NICE diet	Ranked highest for pain improvement. Not superior to habitual diet (RR = 0.72; 65% CI: 0.47–1.10), but superior to sham diet.
RCT [33]	101 (IBS-D)	Low FODMAP vs. TDA	≥50-point reduction in IBS-SSS or adequate symptom relief in 62.7% (FODMAP) vs. 40.8% (TDA); *p* = 0.0448.
RCT [34]	101 (IBS-D)	Low FODMAP vs. TDA	Significant improvement in IBS-SSS for pain intensity (*p* = 0.001) and frequency (*p* = 0.017) for FODMAP vs. TDA.
RCT [35]	104	Low FODMAP vs. sham diet vs. probiotics vs. placebo (2 × 2 factorial)	IBS-SSS was significantly lower for low FODMAP vs. sham (173 ± 95 vs. 224 ± 89; *p* = 0.001), with lower sub scores for pain intensity (*p* = 0.002) and frequency (*p* = 0.001). No difference vs. probiotics and vs. placebo.
RCT [36]	84 (IBS-D)	Low FODMAP vs. mNICE diet	Higher proportion of abdominal pain responders for FODMAP (51%) vs. mNICE (23%); *p* = 0.008.
*Cognitive–behavioral therapy*			
Meta-analysis (42 RCTs) [37]	5220	Brain–gut behavioral therapies (CBT, hypnotherapy, stress management) vs. control	Self-guided/minimal contact CBT reduced pain (RR = 0.71; 95% CI: 0.54–0.95); *p*-score = 0.58; consistently ranked among top interventions.
RCT [38]	558	Internet-/telephone-CBT vs. usual care	Significant reduction in abdominal pain at 12 months (*p* < 0.001); moderate, sustained effect.
RCT [39]	436	Standard CBT vs. minimal-contact CBT vs. education	Both CBT formats improved IBS-SSS; effects were durable at 6 months; NNT = 4–5.
RCT [40]	100	Weekly CBT (10 sessions) vs. usual care	Pain intensity significantly reduced (−1.6 vs. −0.4; *p* = 0.003).
Pharmacological intervention			
*Antispasmodics*			
Meta-analysis (23 RCTs) [41]	2779	Various antispasmodics (e.g., alverine, otilonium, mebeverine, hyoscine) vs. placebo	Significant pain reduction vs. placebo (RR = 1.57; 95% CI: 1.33–1.85); NNT = 5. Broad efficacy across antispasmodic classes.
*Neuromodulators*			
Meta-analysis (7 RCTs) [42]	708	TCAs vs. placebo	Pain persistence reduced (RR = 0.69; 95% CI: 0.54–0.88); low certainty.
Meta-analysis (7 RCTs) [42]	324	SSRIs vs. placebo	Pain persistence reduced (RR = 0.74; 95% CI: 0.56–0.99); very low certainty.
Meta-analysis (2 RCTs) [42]	94	SNRIs vs. placebo	Substantial reduction (RR = 0.22; 95% CI: 0.08–0.59); very low certainty.
Meta-analysis (2 RCTs) [42]	415	Gabapentinoids vs. placebo	No significant difference in abdominal pain.
*Secretagogues and mixed agents*			
Meta-analysis (3 RCTs) [43]	1773 (IBS-C)	Linaclotide vs. placebo	≥30% pain reduction in ≥75% of weeks: RR = 1.58 (95% CI: 1.02–2.46).
Meta-analysis (9 trials) [44]	2309 (IBS-C)	Lubiprostone vs. placebo	Pain reduction at 1 week (combined SMD = 0.55; 95% CI: 0.19–0.91; *p* = 0.003); no effect at 1 or 3 months.
Phase II trial [45]	807 (IBS-D)	Eluxadoline 5–200 mg vs. placebo	Combined response (pain + stool consistency) at week 4: 12.0–13.8% (eluxadoline) vs. 5.7% (placebo); *p* < 0.05.
Phase III trials (IBS-3001, IBS-3002) [46]	2427 (IBS-D)	Eluxadoline 75–100 mg vs. placebo	Combined response: 23.9–29.6% (eluxadoline) vs. 16.2–17.1% (placebo); *p* ≤ 0.01.
*Peppermint oil*			
Meta-analysis (5 RCTs) [47]	357	Peppermint oil vs. placebo	RR = 2.14 (95% CI: 1.64–2.79); significantly more effective than placebo.

Summary of major randomized trials and meta-analyses evaluating interventions for abdominal pain in IBS, including both non-pharmacological (e.g., dietary and psychological) and pharmacological (e.g., antispasmodics, neuromodulators, Secretagogues and mixed agents and peppermint oil) treatments. Key efficacy outcomes are presented. TDA, NICE diet and mNICE diet emphasize eating regular meals, avoiding known dietary triggers, and reducing intake of alcohol and caffeine. Low FODMAP diet consists of the restriction of fermentable oligosaccharides, disaccharides, monosaccharides, and polyols, which are poorly absorbed short-chain carbohydrates found in certain fruits, vegetables, legumes, dairy products, and sweeteners. BGBT: Brain–gut behavioral therapy; CBT: Cognitive–behavioral therapy; IBS: Irritable bowel syndrome; IBS-C: IBS with constipation predominance; IBS-D: IBS with diarrhea predominance; IBS-SSS: IBS Symptom Severity Score; NICE: National Institute for Health and Care Excellence; NNT: Number needed to treat; RCT: Randomized controlled trial; RR: Relative risk; SDM: Standardized difference in mean change; SNRI: Serotonin–norepinephrine reuptake inhibitor; SSRI: Selective serotonin reuptake inhibitor; TCA: Tricyclic antidepressant; TDA: Traditional dietary advice.

**Table 2:** Summary of Clinical Studies on Alverine Citrate/Simeticone in IBS.

Study	Design and Setting	Population	Primary Endpoint	Main Results
Wittmann 2010 [11]	RCT, double-blind, multicenter	409 IBS patients (Rome III)	≥50% reduction in pain VAS (4 weeks)	Responder rate: 46.8% vs. 34.3% (*p* = 0.01); median VAS: 40 mm vs. 50 mm (*p* = 0.047); early benefit from week 2; improved global symptom perception
Ducrotté 2013 [12]	Pragmatic RCT, open-label, primary care	436 IBS patients (IBS-SSS 175–400)	IBS-QoL improvement (6 months)	QoL gain: +13.8 vs. +8.4 points (*p* = 0.0008); ≥50% IBS-SSS reduction: 58.6% vs. 35.9% (*p* = 0.0001); clinical remission: 37.7% vs. 16.0% (*p* < 0.0001); lower healthcare use

## Data Availability

No new data were created or analyzed in this study. Data sharing is not applicable to this article.
